# Examining the Roles of Technology Across the Health Care Journey for Individuals With Obsessive-Compulsive Disorder: Qualitative Interview Study

**DOI:** 10.2196/94674

**Published:** 2026-07-29

**Authors:** Sunidhi Gupta, Lucas Occhino-Moede, Elisa Liu, Thomas Baek, Kaitlyn Sulivan-Pascual, Kendall Phelan, Erica Schug, Megan Mirkis, Tamerlane Visher, Ujjwal Pasupulety, Rachel Carmen Ceasar, Barbara Van Noppen, Adam Charles Frank

**Affiliations:** 1 Keck School of Medicine University of Southern California Los Angeles, CA United States; 2 Dana and David Dornsife College of Letters, Arts and Sciences University of Southern California Los Angeles, CA United States; 3 Department of Psychiatry and Behavioral Sciences Keck School of Medicine University of Southern California Los Angeles, CA United States; 4 Department of Population and Public Health Sciences Keck School of Medicine University of Southern California Los Angeles, CA United States

**Keywords:** obsessive-compulsive disorder, OCD, obsessions, compulsions, technology, qualitative, artificial intelligence, AI, wearable

## Abstract

**Background:**

As digital technologies become increasingly embedded in daily life, their roles in mental health care have expanded and diversified. Digital tools are being explored as interventions for obsessive-compulsive disorder (OCD) across the care continuum, including symptom recognition, access to care, treatment, and self-management. However, there is limited empirical understanding of how individuals living with OCD use digital technologies in situ to navigate their health care journeys or how they envision technology shaping future models of care.

**Objective:**

This study aimed to explore how individuals living with OCD use digital technologies to navigate their health care experiences and to understand the perceived roles of technology across the OCD care continuum.

**Methods:**

We conducted semistructured interviews (N=24) with adults who self-reported a diagnosis of OCD and were recruited through online OCD communities and advocacy networks. Interviews were conducted via Health Insurance Portability and Accountability Act **(**HIPAA)–compliant Zoom between May and December 2024 (median duration 51, IQR 6.25 minutes). Transcripts were coded in Dedoose (version 9.2.22) using a constructivist grounded theory approach. Coding proceeded iteratively through open and focused coding, with theoretical saturation reached after 15 interviews. Constant comparison and analytic memos guided the development of a conceptual framework describing the relationships between technology and OCD health care.

**Results:**

Participants (median age 26, IQR 12.8 years; range 20-64 years; 16/24, 67% female; 7/24, 29% male; 1/24, 4% nonbinary) described technology as integral to 3 aspects of the OCD health care journey. The first aspect was discovery and understanding, in which social media and online content helped participants identify their symptoms, relate them to the diagnosis, and seek care. The second aspect was facilitating OCD health care, in which digital tools such as telehealth platforms, apps, and wearables helped participants bridge gaps in care. The third aspect was imagining the future, in which participants viewed emerging technologies as potential ways to improve symptom recognition and management.

**Conclusions:**

Digital technologies are reshaping how individuals with OCD identify symptoms, engage with treatment, and envision future care delivery. Participants described using online communities, telehealth, and mobile health tools to address barriers across the OCD health care continuum, while emphasizing interest in technologies that provide adaptive and real-time symptom support. These findings underscore the importance of incorporating lived experience into digital mental health innovation and suggest that technology may support a shift toward more continuous, personalized, and accessible OCD care models.

## Introduction

Obsessive-compulsive disorder (OCD) is a psychiatric condition characterized by intrusive thoughts and compulsive behaviors that can significantly impair social and occupational functioning, as well as quality of life [1,2]. Despite the severity of symptoms and the availability of evidence-based treatments such as cognitive behavioral therapy (CBT) and pharmacotherapy, individuals with OCD often face significant barriers to timely and effective care [3]. Studies have found that the average duration between symptom onset and help seeking is nearly 7 years, although delays in accessing care may extend to as long as 17 years [4-6]. Key factors contributing to these delays include a lack of knowledge about OCD and its treatments, as well as the misattribution of symptoms, with many patients unaware that their symptoms are pathological or that effective treatments exist [7,8]. Stigma, shame, and embarrassment further impede disclosure and help seeking. Health system–level barriers, such as limited access to clinicians trained in CBT with exposure and response prevention (ERP), high out-of-pocket costs, and long wait times for specialized care, also contribute to delayed diagnosis and treatment [7-9].

Recently, technology has become increasingly integrated into mental health care, offering novel tools for education, diagnosis, treatment, and long-term management [10,11]. For OCD specifically, a growing body of evidence supports the efficacy of technology-enabled interventions, including but not limited to digital ERP apps, teletherapy, and app-based programs [12-17]. By addressing key barriers such as limited access to trained clinicians, geographic constraints, and financial burden, digital tools are expanding access to evidence-based care [18]. Although wearables and AI systems remain under investigation for continuous monitoring and personalized feedback, early evidence suggests that they show promise for enhancing accessibility and engagement throughout the OCD care continuum [19]. Overall, technology-enabled interventions improve access to evidence-based OCD care, maintain or enhance treatment outcomes, and support patient engagement throughout the health care journey, from symptom recognition to ongoing management [12-17,19].

Although existing research has primarily focused on evaluating the effectiveness of specific digital interventions, less is known about how individuals with OCD experience and engage with technology across the entire health care journey, from symptom recognition and help seeking to diagnosis, treatment, and long-term management. Recent research examining patient preferences for mobile health solutions for OCD suggests that individuals have strong interest in and positive expectations regarding the use of digital tools for symptom monitoring, education, and medication tracking, even among those who are not currently using such tools [20]. Furthermore, our own work has shown how specific features of modern technologies, such as notifications, information provision platforms, gamification metrics, and user interface design, interact with established OCD symptom dimensions, often reinforcing compulsive checking, reassurance-seeking, and ordering behaviors [21]. Together, these findings underscore the growing relevance of digital environments in shaping the lived experience of OCD.

The current study addresses this gap by exploring qualitative accounts from individuals with OCD regarding how diverse technologies shape their lived experiences across the OCD care continuum, including mobile apps, social media, telehealth platforms, wearable devices, and AI-based tools. Findings are organized into three domains reflecting key stages of this journey: (1) *discovery and understanding*, describing how online content facilitates symptom recognition and diagnostic awareness; (2) *facilitating OCD health care*, capturing how technologies support access to and engagement with care; and (3) *imagining the future*, reflecting participants’ aspirations for more responsive and personalized technological support.

## Methods

### Study Design

This study draws on a phenomenological approach to explore how people with OCD describe their interactions with technology in the context of health care. Data collection and analysis were informed by constructivist grounded theory, which conceptualizes knowledge as coconstructed between researchers and participants and emphasizes iterative, comparative analysis to build theory grounded in the data [22]. We used the Consolidated Criteria for Reporting Qualitative Research (COREQ) checklist to structure reporting and to ensure transparency regarding the research team, study design, data collection, and analytic procedures [23] (Multimedia Appendix 1).

### Recruitment and Sample

Recruitment processes and participants are identical to those described by Occhino-Moede et al [21]. Briefly, participants were recruited via convenience sampling from May to December 2024. Targeted outreach used online communities focused on OCD education, advocacy, and peer support. Recruitment notices were distributed through platforms affiliated with the International OCD Foundation and through OCD-focused research participation registries maintained by the laboratory. These online spaces represent commonly used digital environments where individuals with OCD seek educational resources and treatment information.

Recruitment posts consisted of a standardized announcement detailing the study purpose, eligibility criteria, participation expectations, and compensation. Those interested in participating were directed to contact the research team via email and to complete a REDCap (Vanderbilt University) screening survey that collected basic demographic information. Survey responses were sequentially reviewed in order of submission. Individuals meeting the eligibility criteria were invited to participate in a virtual onboarding session to confirm screening responses, discuss study procedures, and complete informed consent.

Inclusion criteria included a self-reported age of ≥18 years and a diagnosis of OCD, and a total of 27 participants completed the consent process and virtual interview. All interviews were reviewed by study personnel trained in the phenomenology of OCD to confirm that participants demonstrated knowledge of and experience with OCD. Three interviews were excluded due to credible concerns regarding participants’ eligibility (eg, no clear indication of an OCD diagnosis or suspected repeat participation by a single individual). We implemented procedures to mitigate this risk, including requiring participants to keep their cameras on during interviews and conducting a brief discussion of their OCD symptoms before proceeding with the interview. No participants withdrew or declined participation after enrollment. A total of 25.9% (7/27) participants had prior contact with laboratory personnel through other studies. Sample characteristics are summarized in Multimedia Appendix 2.

### Research Team and Procedures

The study was conducted by a multidisciplinary team led by the principal investigator (AF, psychiatrist). All interviewers completed a structured 6-week qualitative training and participated in ongoing supervision (including review of the interview guide, observation by senior interviewers, and postinterview debriefings).

We used a semistructured interview guide to elicit participants’ experiences with digital technologies used for health and health care, with a focus on OCD-related care. An initial version was piloted within the research team and iteratively refined during data collection in response to interviewer reflections and emerging analytic insights, consistent with constructivist grounded theory.

Interviews were conducted remotely via Health Insurance Portability and Accountability Act (HIPAA)–compliant Zoom (Zoom Communications Inc) between May 16, 2024, and December 20, 2024. With participant permission, sessions were audio- and video-recorded and auto-transcribed within Zoom. Research assistants reviewed transcripts for accuracy and deidentification.

The first 5 interview transcripts were independently open-coded line by line by pairs of researchers, who met to reconcile interpretations and develop an initial codebook. All transcripts were then imported into Dedoose (version 9.2.22; SocioCultural Research Consultants LLC) and double-coded by 2 team members. Coder pairs met to resolve discrepancies and refine code definitions through consensus. Theoretical saturation was achieved by the 15th interview.

Details of the procedures, including the demographics of the research team, the sample interview guide, data collection and management, and saturation procedures, are described in detail elsewhere and in Multimedia Appendices 3-6 [21].

### Theory Development

Analysis began with a targeted review of excerpts coded with the conceptually pertinent code (ie, “Exploring the role(s) of technology and Mental Healthcare”) and its co-occurrences with other codes. These excerpts were used to write analytical memos, which guided weekly team discussions and supported theoretical elaboration. Through this iterative process, we developed a conceptual framework describing the major domains of technology use across the OCD health care journey and the ways participants understood these interactions. Negative cases, notably in relation to online platforms and social media, were identified, and our conceptual framework was updated to account for these findings [24].

### Ethical Considerations

All study procedures were reviewed and approved by the University of Southern California Institutional Review Board (UP-23-01094). Prospective participants received written information describing the study purpose, procedures, potential risks and benefits, and the voluntary nature of participation. Study staff reviewed this information during a videoconference consent visit and addressed any questions before obtaining informed consent. Participants were informed that they could decline to answer any question or withdraw from the study at any time without penalty or loss of benefits. Interview data were deidentified prior to analysis, and all files were stored on secure, access-restricted servers to protect confidentiality. Participants received a Tango gift card valued at US $50 as compensation after completing the interview.

## Results

### Participants

A description of the participants (N=24) is provided (Multimedia Appendix 2). Briefly, the median age was 26.2 (IQR 12.8) years; the age range was 20.1 to 63.6 years. The sample comprised 7 (29.2%) men, 16 (66.7%) women, and 1 (4.2%) nonbinary individual. No participants refused to participate in the study or withdrew their participation after enrollment.

### Coding Tree and Domains

A coding tree with 5 parent codes and 7 child codes was developed during analysis (Multimedia Appendix 5). Among these, the parent code “Exploring the role(s) of technology and Mental Healthcare” was most central to addressing the research question and guided the organization of the findings, with additional codes examined for co-occurrence with this central code. From this coding structure, we identified three domains reflecting key intersections of technology and the OCD health care journey: (1) discovery and understanding, (2) facilitating OCD health care with technology, and (3) imagining the future.

### Discovery and Understanding

Across interviews, participants described their engagement in online spaces as integral to their experiences of identifying with and learning about OCD. Digital communities helped them recognize their symptoms, seek diagnosis, and feel validated through others’ narratives:

I’ve met friends that I’m in Discord servers in, and a lot of them have OCD. And actually, before I got diagnosed, they really helped because they would tell me their symptoms and they were diagnosed already. And I’d be like, “Oh wait, I relate to that [or] oh wait, I have that.” And so it helped push me into seeking diagnosis.

Participant 1

Like participant 1, participant 2 described how identifying symptoms through social media helped them explore this diagnosis with their therapist:

When I wasn’t sure if I had OCD or not, seeing all the symptoms [on Reddit posts] [made me realize] I probably do [have OCD], and that’s why I brought it up to my therapist, who diagnosed me, it was because I was like pretty convinced I had it from reading through all those community threads and being like, “This does sound like what is going on [with me].”

Participant 2

Following diagnosis, many participants continued to rely on social media and personal accounts to further their understanding of OCD:

[When I was] in therapy...I was looking at OCD Reddit threads, not [just wanting] academic [information] but also personal experiences, and hearing [how] other people’s experiences were similar to [mine]. Even now, I follow some social media OCD-related pages, just for good information.

Participant 3

Like participant 3, participants used social media pages focused on OCD content for continued support and education:

I use YouTube for encouragement. I found this comedian [with] OCD who posts a lot about OCD. So I use that for educational content. Same with TikTok - [many] very specific OCD themes, just don’t really have relatable articles for them, so I just will go on TikTok and watch other OCDers talk about it.

Participant 4

Beyond increasing understanding, participants emphasized that connecting with others’ stories online provided validation, solidarity, and emotional relief:

What I read from other people...you feel very alone or think your thoughts are crazy...So I feel like [it] is reassuring to find other people [and realize], “I’m not the only one with this.”

Participant 3

It makes me feel less alone in that sphere. And less crazy, honestly, because people have really preconceived notions about OCD.

Participant 5

Some participants shared a preference for resources that were more clinical or educational in nature:

[The online OCD community] has allowed me to learn that there are some different subtypes of OCD, so that’s been helpful and [it has also allowed me to] feel less isolated in my symptoms as well as empower myself to seek other healthcare providers. But I’m talking about the International OCD foundation – those Instagram pages that are not run by content creators, but by nonprofit organizations, or medical organizations.

Participant 6

Participants also described engaging with psychoeducational content through podcasts, which were described as supporting symptom recognition, normalizing experiences, and fostering community connection:

I forgot [to mention] podcasts like FearCast...And Kimberly Quinlan has a great podcast for, like, people with OCD, I think those sorts of resources are awesome for community building and just kind of understanding symptoms and patterns.

Participant 7

Although the availability of OCD-related resources was helpful for most participants, some also noted drawbacks associated with digital engagement. For instance, participant 4 described how these platforms could reinforce checking behavior by facilitating online reassurance-seeking:

...in terms of the actual symptoms of my OCD, checking is a big thing. Like I Google things, I go on Reddit, I try to find similar situations to whatever my brain is worried about so that I can get reassurance.

Participant 4

Similarly, 1 participant articulated the triggering role of OCD content despite the benefits of the online OCD community:

Once I found out that there was an OCD subreddit, which is a forum where you can talk to other people about this specific topic, I was really excited to have that kind of community. But I did end up having to unsubscribe from that after a certain point because it can get really triggering when people are sharing their in-depth obsessions and compulsions.

Participant 8

Overall, although online narratives and resources helped participants name their symptoms, seek a diagnosis, and feel less alone, they also introduced risks, highlighting the dual role of digital platforms in both supporting and complicating the experiences of individuals with OCD.

### Facilitating OCD Health Care With Technology

Across interviews, participants described digital tools as affording concrete ways of accessing, sustaining, and extending OCD care. They spoke about using technology to reach clinicians, obtain support when traditional options felt inaccessible or invalidating, supplement ERP and other OCD-specific treatments, and track bodily signs linked to symptoms.

Participants frequently described difficulties accessing mental health care and OCD-specific care due to geography, transportation, or uncertainty about where to find specialized clinicians. For some, following OCD-focused organizations online provided a starting point for identifying clinicians and services. One participant described how a national organization’s social media accounts and website helped them locate potential clinicians:

I think that the International OCD Foundation have their links in their (social media) bio. And I remember going to their website...that was helpful to review a list of providers.

Participant 6

Another participant described how transitioning to online psychiatry allowed them to re-engage in treatment by providing greater access to and easier “filtering” of health care providers:

Because I’m in college, I’ve had better luck just seeing a psychiatrist online. And, frankly, I stopped seeing a psychiatrist for a long time because I’ve had some bad experiences...It took me a really long time to find a psychiatrist that I would actually see, so doing it online was really beneficial because I could filter through [different provider options].

Participant 9

Although participants more frequently described technology as facilitating access to human therapists and psychiatrists, 1 participant described relying heavily on an AI tool designed for mental health support. They contrasted a prior diagnostic encounter during which they felt dismissed with their ongoing use of the AI tool for mental health concerns:

When I was diagnosed with severe OCD, if [healthcare provider] had just asked me a couple of follow-up questions, that would have been nice. Even with the state of training data not being perfect, I cannot foresee an AI ever dismissing me like that. I talk to AI a lot about mental health stuff, and it’s always miles ahead of where my healthcare providers have been in terms of understanding the nuances of mental illness and knowing the best way to talk about things...Ideally, I wouldn’t even have to talk to a human, but I don’t think we’re quite there yet.

Participant 8

Participants also described using technology to enrich their OCD-specific treatment, particularly ERP. One participant explained how an app helped them organize exposures and rate anxiety levels associated with resisting compulsions:

They have a great feature...where you can put in your obsession, specific compulsions, and what your level of anxiety would be if you didn’t perform those compulsions, 1 through 10. It’s set up to have you rank what gives you the most anxiety...Then you can choose one of [the exposures] and it gives you a timer [to complete it]. It lets you easily organize your obsessions and compulsions.

Participant 4

Participants reported using these tools between sessions to support ongoing ERP work rather than as a replacement for therapist-guided treatment.

A subset of participants described using wearable devices and health apps to track sleep, heart rate, and other physiological indicators that they associated with anxiety, mood changes, or compulsions. One participant explained how they occasionally reviewed data from a wearable device with their psychiatrist to help make sense of symptom fluctuations:

Sometimes, yeah, especially in times where I know that I was super anxious or something, and I have biological proof of that...Also sleep, I think sleep is a big trigger for me for mood changes and anxiety and just kind of an uptick in overall mental health symptoms. So I’m able to look and see how much sleep I got the night before and what was the quality of my sleep. I see, “Oh, I didn’t get a lot of sleep. My sleep quality was really low. That’s probably why I’m feeling this way and experiencing these symptoms.”

Participant 9

Another participant described using a Fitbit to monitor sleep and heart rate and observed that physiological responses sometimes coincided with stress, compulsions, or tics:

Definitely knowing what my sleep was like was the most interesting thing for me. And also, it was interesting to see my heart rate...Sometimes I get overheated, like I have a physical response...if I get really stressed and overwhelmed by either a compulsion or tic sometimes...I guess just knowing like certain, um...is the word biometrics? Yeah, I guess that was the benefit.

Participant 1

Overall, participants described technology as providing options for accessing OCD care that felt responsive to their needs and as supporting ongoing care through ERP and symptom tracking.

### Imagining the Future

Participants described ways in which technology could better support ongoing challenges in OCD care. These reflections frequently centered on technologies that could respond dynamically to symptoms, improve diagnostic and clinical processes, and better accommodate the cognitive and sensory needs associated with OCD.

Many participants envisioned wearable or mobile technologies capable of recognizing symptom escalation and providing real-time support. These imagined tools were often described as helping interrupt obsessive or anxiety loops in real time:

I get really, really anxious and I start to get kind of out of touch with reality. So if I had a watch that could tell me when I was getting out of touch with reality, that would be awesome.

Participant 2

Similarly, participants expressed interest in tools that could provide interactive feedback or encouragement during moments of distress:

Maybe [a wearable device] app could be interactive... [individuals with OCD] could get advice or feedback, or support, or encouragement on the spot...when they may be facing that thing that they’re worried about or fear.

Participant 10

Beyond moment-to-moment symptom management, some participants described how technology could address challenges in the diagnostic process. These participants discussed how behavioral or use data might help identify OCD patterns that are difficult to capture during traditional clinical encounters:

Tracking what people do and how they interact in their day-to-day — those things that are really hard to catch...There’s such a crisis in diagnostic capacity...I’m really interested in how data could point to something a little bit more reliably identifiable.

Participant 7

In addition to expanding care access and assessment, participants emphasized design features that would make digital tools more compatible with OCD symptoms. One participant described how excessive notifications from current technologies could worsen checking behaviors and suggested simplified or controlled notification systems:

I would be really interested in [wearable technology] if it doesn’t have all the capabilities of a smartphone...maybe there is a way to silence all notifications and the only notification you get is if a person is using it for OCD reasons...because [current wearable technology] bombards you with a lot.

Participant 11

Across interviews, participants described future technologies as tools that could support symptom interruption, assist in the clinical identification of OCD, and expand treatment possibilities while remaining sensitive to the cognitive and sensory demands associated with OCD.

## Discussion

### Summary of Findings

This study explored how individuals with OCD engage with technology throughout their health care journey, from symptom recognition to ongoing management. Three main findings emerged in relation to our study objectives: (1) online content and peer communities generally facilitated early symptom recognition and helped reduce stigma; (2) digital tools, including telehealth and mobile apps, supported access to and continuity of OCD care; and (3) participants expressed interest in future technologies capable of providing real-time symptom monitoring, personalized feedback, and adaptive support during periods of escalating distress.

### Findings in Context

The first finding centers on the critical role of online content and peer communities in helping participants recognize OCD symptoms and reduce stigma before formal diagnosis. Participants frequently described anecdotal content encountered through platforms such as Reddit (Reddit Inc), TikTok (ByteDance), and YouTube (Google) as a turning point in their self-identification with OCD. Recent literature suggests that younger individuals report shorter delays between symptom onset and OCD recognition, which may reflect increased access to digital mental health communities and symptom-focused online content [7]. Participants described both anecdotal narratives and formal psychoeducational materials as reducing fear and uncertainty surrounding OCD while simultaneously promoting and facilitating help-seeking behaviors. However, social media content can both inform and mislead; although sharing experiences with peers fosters understanding, the spread of misinformation and stereotype-driven content, particularly on TikTok, can reinforce misconceptions about OCD [25]. Together, these findings suggest that digital mental health interventions may benefit from incorporating narrative- and community-informed approaches while ensuring the integration of clinically accurate, evidence-based information to support early symptom recognition and informed help seeking.

Notably, 1 participant commented on the positive role that AI played in their mental health journey and expressed the view that these systems were not only beneficial but also surpassed the capabilities of human clinicians. In community samples, a variety of opinions exist about the integration of AI into mental health care and support, although positive and optimistic views have been expressed by both individuals with lived experience and health care providers [26]. The participant in our study also expressed a preference to fully engage with AI systems and not speak with humans for mental health care. This lack of connection to human clinicians was a concern for most individuals with lived experience in a community sample [26] and raises important questions regarding the intended and unintended uses of AI in supporting and treating individuals with mental health concerns [27]. Issues that the field is beginning to address—and for which ongoing qualitative and quantitative studies are needed—include the veracity of AI-generated content [28,29], algorithmic bias in AI models and approaches to mitigating such bias [30,31], and safety considerations related to imminent risk in mental health situations [32-34]. The possibility of AI systems supporting human-delivered care, for instance, by aiding a therapist in generating an exposure hierarchy with a patient in the context of ERP, may strike a middle ground between entirely AI-based care and the refusal of patients and clinicians to engage with these systems.

Our second theme highlights the role of digital tools in facilitating access to and continuity of OCD care. Participants emphasized how remote therapy allowed them to overcome geographic and logistical barriers, remain connected to trusted clinicians across relocations, and sustain long-term therapeutic relationships. Existing outcome-based literature suggests that, for individuals with OCD who experience avoidance behaviors or fears of leaving their home, telehealth may offer a critical and accessible alternative to in-person visits [10,11,15]. These findings align with additional evidence supporting remote ERP and internet-based CBT as effective first-line treatments, particularly for individuals facing mobility or anxiety-related barriers to care [13-16]. Beyond improving access to clinicians, participants described using digital tools to extend treatment beyond traditional clinical encounters. Some reported using structured mobile apps to reinforce ERP skills between sessions, which were generally viewed as helpful adjuncts to therapist-guided treatment. Participants also described using wearable devices and health apps to monitor physiological indicators such as sleep quality and heart rate, which they used to contextualize symptom fluctuations and support self-management strategies. Overall, these findings suggest that individuals with OCD are using digital technologies in both clinical and novel ways to actively bridge gaps in their care, reflecting a desire for more continuous and responsive symptom management than currently available.

Our third finding further highlights participants’ desire for future technologies to address ongoing gaps in OCD care. Particularly, participants frequently described an interest in technologies capable of providing real-time symptom monitoring, personalized feedback, and proactive support during periods of escalating distress. Wearable devices and interactive mobile tools were often imagined as mechanisms for detecting physiological or emotional changes and prompting coping strategies, reflecting a desire for interventions that respond dynamically to symptom fluctuations. Although the current body of research in the area of OCD and biomarkers is examining diagnosis, phenotypic characterization, and treatment response prediction, our findings suggest that individuals with OCD are additionally hopeful that these technologies can be leveraged to provide real-time symptom support and adaptive, patient-responsive intervention strategies.

### Limitations

These findings should be considered within the context of several study limitations. Because participants were recruited through online platforms, the sample may reflect individuals who are already engaged with digital tools and who hold more favorable or critical views of technology than those with limited access. Additionally, this approach to recruitment may underrepresent minoritized communities with limited access to technology or treatment resources in general, a recognized challenge within the broader OCD community [35,36]. Overall, our study does not capture the experiences of individuals with OCD who have limited digital access, are less engaged with online communities and platforms, or come from socioeconomically disadvantaged backgrounds. Targeted inclusion of these voices and perspectives in future studies will provide a more complete perspective on the lived experience of the OCD community.

Although our screening and enrollment process attempted to ascertain familiarity with lived experience consistent with OCD, all participants self-identified as having OCD, and we did not conduct structured diagnostic interviews to assess for OCD. Thus, some participant narratives may overlap with symptoms of related conditions, such as social anxiety disorder or generalized anxiety disorder. Furthermore, we did not screen for or explicitly ask about co-occurring conditions such as attention-deficit/hyperactivity disorder (ADHD) or depression, and their prevalence rates in adults with OCD are approximately 13% and 32%, respectively [37,38]. Thus, the narratives elicited from our participants should be viewed as reflective of this sample’s experiences with symptoms of OCD and other conditions potentially present. Nevertheless, self-reported diagnosis is established in qualitative mental health research spanning conditions including depression, OCD, and bipolar disorder [39-42]. In line with these studies, our sampling strategy is consistent with the goal of experiential inquiry, although cautious interpretation regarding the boundaries between OCD and other anxiety disorders is important.

The sample was modest in size and skewed toward younger adults, which may underrepresent the perspectives of older individuals or those less comfortable with digital engagement. Finally, as with other qualitative research, the analytic framework reflects an interpretation coconstructed by participants and the research team. Although we sought to enhance trustworthiness through reflexive discussion and consensus coding, the themes presented here should be understood as representing one perspective on the role of technology in OCD care.

### Conclusions

This study demonstrates that digital technologies are not simply adjunct tools in OCD care but are increasingly shaping how individuals recognize symptoms, access treatment, and imagine future therapeutic possibilities. Participants described using online communities, telehealth platforms, mobile apps, and emerging digital tools to address gaps across the OCD health care journey, often fulfilling needs that traditional care models have struggled to meet. These findings suggest that effective digital mental health innovation for OCD will require approaches that integrate evidence-based clinical care with patient-centered design, lived experience narratives, and continuous symptom support. As digital technologies continue to evolve, incorporating patient perspectives into the development and implementation of these tools will be critical to ensuring that technology enhances accessibility, therapeutic engagement, and long-term outcomes for individuals living with OCD.

## Data Availability

The datasets generated or analyzed during this study are available from the corresponding author on reasonable request.

## Appendix

Multimedia Appendix 1 COREQ 32-item checklist.

Multimedia Appendix 2 Participant demographic information (N=24).

Multimedia Appendix 3 Demographic and training characteristics of the research team members and their involvement in conducting qualitative interviews.

Multimedia Appendix 4 Sample interview guide.

Multimedia Appendix 5 Coding tree.

Multimedia Appendix 6 Saturation tracking using the global “New Finding” code.

## Abbreviations

ADHD: attention-deficit/hyperactivity disorder
CBT: cognitive behavioral therapy
COREQ: Consolidated Criteria for Reporting Qualitative Research
ERP: exposure and response prevention
HIPAA: Health Insurance Portability and Accountability Act
OCD: obsessive-compulsive disorder
