# Supplementary material for: Examining the Roles of Technology Across the Health Care Journey for Individuals With Obsessive-Compulsive Disorder: Qualitative Interview Study
Source: J Med Internet Res. 2026 Jul 29;28:e94674. doi: 10.2196/94674 (PMC13469831; doi:10.2196/94674)
Supplement: Supplementary file 1 [file jmir_v28i1e94674_app1.docx]

| **Item Number** | **Guide Questions/Description** | | | | **Reported on Page #** |
| --- | --- | --- | --- | --- | --- |
| **Domain 1: Research team and reflexivity** | | | | | |
| **Personal Characteristics** | | | | | |
| 1. Interviewer/ facilitator | Which author/s conducted the interview or focus group? | | | | Appendix 3 |
| 2. Credentials | What were the researcher’s credentials? E.g., PhD, MD | | | | Appendix 3 |
| 3. Occupation | What was their occupation at the time of the study? | | | | Appendix 3 |
| 4. Gender | Was the researcher male or female? | | | | Appendix 3 |
| 5. Experience and training | What experience or training did the researcher have? | | | | Appendix 3, Pg 3 |
| **Relationship with participants** | | | | | |
| 6. Relationship established | Was a relationship established prior to study commencement? | | | | Pg 3 |
| 7. Participant knowledge of the interviewer | What did the participants know about the researcher? e.g. personal goals, reasons for doing the research? | | | | Pg 3 |
| 8. Interviewer characteristics | What characteristics were reported about the interviewer/facilitator? e.g. Bias, assumptions, reasons and interests in the research topic | | | | Pg 3 |
| **Domain 2: study design** | | | | | |
| **Theoretical framework** | | | | | |
| 9. Methodological orientation and Theory | | | What methodological orientation was stated to underpin the study? e.g. grounded theory, discourse analysis, ethnography, phenomenology, content analysis | | Pg 2 |
| **Participant selection** | | | | | |
| 10. Sampling | | | How were participants selected? e.g., purposive, convenience, consecutive, snowball | | Pg 2-3 |
| 11. Method of approach | | | How were participants approached? e.g., face-to-face, telephone, mail, email | | Pg 2-3 |
| 12. Sample size | | | How many participants were in the study? | | Pg 2-3 |
| 13. Non-participation Setting | | | How many people refused to participate or dropped out? Reasons? | | Pg 2-3 |
| 14. Setting of data collection | | | Where was the data collected? e.g., home, clinic, workplace | | Pg 2-3 |
| 15. Presence of nonparticipants | | | Was anyone else present besides the participants and researchers? | | Pg 2-3 |
| 16. Description of sample | | | What are the important characteristics of the sample? e.g. demographic data, date | | Pg 4 |
| **Data collection** | | | | | |
| 17. Interview guide | | Were questions, prompts, and guides provided by the authors? Was it pilot tested? | | Appendix 4 | |
| 18. Repeat interviews | | Were repeat interviews carried out? If yes, how many? | | Pg 3 | |
| 19. Audio/visual recording | | Did the research use audio or visual recording to collect the data? | | Pg 3 | |
| 20. Field notes | | Were field notes made during and/or after the interview or focus group? | | N/A | |
| 21. Duration | | What was the duration of the interviews or focus group? | | Pg 3 | |
| 22. Data saturation | | Was data saturation discussed? | | Pg 3 | |
| 23. Transcripts returned | | Were transcripts returned to participants for comment and/or correction? | | N/A | |
| **Domain 3: analysis and findings** | | | | | |
| **Data analysis** | | | | | |
| 24. Number of data coders | | How many data coders coded the data? | | Pg 3 | |
| 25. Description of the coding tree | | Did the authors provide a description of the coding tree? | | Appendix 5 | |
| 26. Derivation of themes | | Were themes identified in advance or derived from the data? | | Pg 3-4 | |
| 27. Software | | What software, if applicable, was used to manage the data? | | Pg 3 | |
| 28. Participant checking | | Did participants provide feedback on the findings? | | Pg 3 | |
| **Reporting** | | | | | |
| 29. Quotations presented | | Were participant quotations presented to illustrate the themes/findings? Was each quotation identified? e.g., participant number | | Pg 5-11 | |
| 30. Data and findings consistent | | Was there consistency between the data presented and the findings? | | Pg 5-11 | |
| 31. Clarity of major themes | | Were major themes clearly presented in the findings? | | Pg 5-11 | |
| 32. Clarity of minor themes | | Is there a description of diverse cases or a discussion of minor themes? | | Pg 5-11 | |
