# Supplementary material for: Examining the Roles of Technology Across the Health Care Journey for Individuals With Obsessive-Compulsive Disorder: Qualitative Interview Study
Source: J Med Internet Res. 2026 Jul 29;28:e94674. doi: 10.2196/94674 (PMC13469831; doi:10.2196/94674)
Supplement: Supplementary file 2 [file jmir_v28i1e94674_app2.docx]

Appendix 2. Participant demographic information (N=24).

| Median Age, years (range, IQR) | 26.2 (20.1 - 63.6, 12.8) |
| --- | --- |
| Gender |  |
| Man | 7 |
| Woman | 16 |
| Non-Binary | 1 |
| State |  |
| California | 14 |
| Pennsylvania | 3 |
| Illinois | 2 |
| Ohio | 2 |
| Florida | 1 |
| Maryland | 1 |
| Virginia | 1 |
