# Supplementary material for: Examining the Roles of Technology Across the Health Care Journey for Individuals With Obsessive-Compulsive Disorder: Qualitative Interview Study
Source: J Med Internet Res. 2026 Jul 29;28:e94674. doi: 10.2196/94674 (PMC13469831; doi:10.2196/94674)
Supplement: Supplementary file 3 [file jmir_v28i1e94674_app3.docx]

Appendix 3. Demographic and training characteristics of the research team members and their involvement in conducting qualitative interviews.

| Initials | Education/Occupation | Gender | Conducted Interviews |
| --- | --- | --- | --- |
| ACF | MD, PhD | Man | Yes |
| LO | Medical student | Non-binary | Yes |
| EL | Undergraduate student | Woman | Yes |
| KSP | Undergraduate student | Woman | Yes |
| TV | Medical student | Man | Yes |
| MM | Undergraduate student | Woman | Yes |
| TBAE | Undergraduate student | Man | Yes |
| ES | Undergraduate student | Woman | Yes |
| KP | Medical student | Woman | No |
| HW | Medical student | Man | No |
| DM | Medical student | Man | No |
| UP | Graduate student | Man | No |
| NB | Undergraduate student | Woman | No |
| TBAI | Graduate student | Man | No |
| ZE | Undergraduate student | Woman | No |
| JK | Undergraduate student | Woman | No |
| DS | Medical student | Man | No |
| RC | PhD | Woman | No |
| BVN | PhD | Woman | No |
