# Supplementary material for: Examining the Roles of Technology Across the Health Care Journey for Individuals With Obsessive-Compulsive Disorder: Qualitative Interview Study
Source: J Med Internet Res. 2026 Jul 29;28:e94674. doi: 10.2196/94674 (PMC13469831; doi:10.2196/94674)
Supplement: Supplementary file 4 [file jmir_v28i1e94674_app4.docx]

**SAMPLE INTERVIEW QUESTIONS**

- Walk me through how you use smartphone apps on a typical day? (IF NO CURRENT/PAST USE, SKIP TO BOTTOM)
- Tell me about your experience using apps or smartphone features for HEALTHCARE?
  - Physical health?
  - Mental health?
  - For OCD?
  - How about apps in the past (IF NOT CURRENTLY USING)?

IF HISTORY OF USE (current or past):

- What were your **motivations** to start using? To stop using？
- What **benefits** have you experienced?
- What **barriers, difficulties** do you have with using apps?
- What **concerns** do you have?
- Have your **usage patterns** changed?
- What caused these changes?
- How did you **learn about** this app?
  - Describe any conversations you’ve had about this app.
- How has your smartphone and apps **interacted with your OCD**?
  - PROBES: change in **symptom level**, change in **symptom frequency**, change is **type of OCD** symptoms

IF NO HISTORY OF USE:

- Are there any **reasons for not trying** them?
- "Can you share any insight you have about how other people with OCD are using smartphone apps for their health?"

- Walk me through how you use wearable devices on a typical day? (IF NO CURRENT/PAST USE, SKIP TO BOTTOM)
- Tell me about your experience using wearable devices for healthcare?
  - Physical health?
  - Mental health?
  - For OCD?
  - How about devices in the past (IF NOT CURRENTLY USING)?

IF HISTORY OF USE (current or past):

- What were your **motivations** to start using? To stop using？
- What **benefits** have you experienced?
- What **barriers, difficulties** do you have with using wearables?
- What **concerns** do you have?
- Have your **usage patterns** changed?
  - What caused these changes?
- How did you **learn about** this wearable?
  - Describe any conversations you’ve had about this wearable.
- How has the wearable device **interacted with your OCD**?
  - PROBES: change in **symptom level**, change in **symptom frequency**, change is **type of OCD** symptoms

IF NO HISTORY OF USE:

- Are there any reasons for not trying them?
- What do you know about how other people with OCD are wearable devices for their health?

- Are there other forms of technology that you feel have interacted with your OCD? In what format? (**work communications, social media, internet,** e-system, screens, etc.)
- "How would you feel if your doctor or therapist used AI to adjust your medication or guide therapy?"
- “What type of engagement in the OCD community has technology allowed you?”
- Final thoughts + Questions for us + Additional questions?
