# Supplementary material for: Examining the Roles of Technology Across the Health Care Journey for Individuals With Obsessive-Compulsive Disorder: Qualitative Interview Study
Source: J Med Internet Res. 2026 Jul 29;28:e94674. doi: 10.2196/94674 (PMC13469831; doi:10.2196/94674)
Supplement: Supplementary file 6 [file jmir_v28i1e94674_app6.docx]

Appendix 6: Saturation Tracking Using the Global “New Finding” Code

| Interview # | "new finding" applications |
| --- | --- |
| 1-5 | N/A* |
| 6 | 0 |
| 7 | 0 |
| 8 | 2 |
| 9 | 1 |
| 10 | 0 |
| 11 | 1 |
| 12 | 3 |
| 13 | 0 |
| 14 | 0 |
| **15** | **1** |
| 16 | 0 |
| 17 | 0 |
| 18 | 0 |
| 19 | 0 |
| 20 | 0 |
| 21 | 0 |
| 22 | 0 |
| 23 | 0 |
| 24 | 0 |

Appendix 6 summarizes use of the global “New Finding” code, applied when data relevant to the research questions were not captured by existing codes. The absence of new applications after interview 15 indicates that additional data were increasingly redundant with previously coded material, marking the point of inductive thematic saturation.

**Interviews 1–5 were conducted during the open-coding phase, when all relevant data were captured through initial code generation rather than through the global “New Finding” code.*
